# Equity at the point of care: auditing AI-supported resource allocation in obstetric emergencies

**DOI:** 10.3389/fpubh.2026.1774367

**Published:** 2026-03-03

**Authors:** Fan Gao, Danli Xie

**Affiliations:** 1 West China Second University Hospital, Sichuan University, Chengdu, China; 2 Key Laboratory of Birth Defects and Related Diseases of Women and Children, Ministry of Education, Sichuan University, Chengdu, China

**Keywords:** avoidable delay, clinical decision support, equity audit, obstetric emergencies, postpartum hemorrhage, quality improvement, resource allocation

## Abstract

Equity in artificial intelligence-supported obstetric emergency care should be assessed as a service outcome, not as a model property, because preventable harm is mediated through operational delay, escalation, and resource contention. This perspective synthesizes implementation-relevant literature and quality and safety principles to propose a practical approach for translating equity from an abstract aspiration into auditable operations. We argue for using “avoidable delay” as a shared denominator across emergencies (such as postpartum hemorrhage, hypertensive crises, and obstetric sepsis) and for evaluating equity across the full chain from risk detection to resource delivery. We propose a minimum fairness audit set that can be captured largely from routine timestamps and logs: consistency of triggering across comparable presentations; timeliness of first response and definitive treatment; readiness of critical resources (blood products at bedside, operating room access and anesthesia start, and monitored-bed availability); completion of escalation and transfer steps; and structured documentation of overrides, missing data, and exception reasons. We further outline governance requirements—clear cross-service accountability, change control with re-audit after threshold or workflow modifications, and patient-facing transparency—so that equity is treated as an accountable, measured, and managed risk item within routine quality improvement rather than a one-time publication metric. In this perspective, “AI-supported” is used as a pragmatic umbrella to encompass deployed algorithmic decision-support systems at the point of care, including static rules-based early warning triggers, machine-learning risk scores, and operational routing/queuing engines; the Minimum Fairness Audit Set (MFAS) audits the service-chain consequences of any such trigger when it is coupled to an executable pathway with auditable timestamps.

## 1 Introduction

### 1.1 Setting and paradox

In obstetric emergencies, minutes matter because outcomes depend on time-critical access to blood products, operating room availability, anesthesia support, intensive care unit (ICU) capacity, interfacility transfer, and high-acuity monitoring (1). Protocols for postpartum hemorrhage, hypertensive crises, and maternal sepsis are intended to standardize recognition and escalation (2–5). However, comparable clinical risk does not consistently translate into comparable speed or intensity of response. Queues and handoffs—triage thresholds, paging cascades, transport logistics, and competing cases—can delay definitive care even when the diagnosis is clear (1). The paradox is that many gaps reflect workflow design and access constraints rather than missing medical knowledge (3, 4).

### 1.2 Author stance

Equity in artificial intelligence (AI)-supported obstetric emergency care is not a model attribute (e.g., area under the receiver operating characteristic curve [AUC] or calibration); rather, it is whether deployment translates risk signals into timely escalation, resource readiness, and safer disposition for patients presenting with similar risk (6, 7). In practice, deployed tools fail at three predictable breakpoints: a trigger that is not acted on, an action that does not mobilize resources, and resources that arrive too late to alter outcomes (7). Audits that end at model performance can therefore validate a statistically strong tool while allowing inequity to be produced by unclear ownership, capacity bottlenecks, and fragmented handoffs (6). Evaluation should follow the chain of care, quantify step-specific delays and denials, and test whether breakdowns and prolonged intervals cluster within particular groups and contexts (7).

### 1.3 Contribution and roadmap

This perspective provides three deliverables: (i) a service-chain framework that specifies where inequity can emerge after deployment; (ii) a Minimum Fairness Audit Set (MFAS) dashboard designed to be low-burden and auditable; and (iii) a worked application in postpartum hemorrhage (PPH), hypertensive crises/eclampsia, and obstetric sepsis (1, 3, 4). We use World Health Organization pathway standardization as an anchor to define expected trigger-to-treatment transitions and then generalize the audit logic to adjacent obstetric emergencies without overextending what guidelines can legitimately support (1).

## 2 Methods

Search strategy and selection criteria. PubMed and Scopus databases were searched on 30 December 2025 (English; 2019/01/01–2025/10/18) using three AND-combined blocks: obstetric emergencies/maternal early warning (postpartum/obstetric hemorrhage, eclampsia/hypertensive crisis, maternal/obstetric sepsis, and maternal early warning/early warning score/system); AI−/clinical decision support-enabled triggers (clinical decision support, alerts, risk scores, machine learning/artificial intelligence, and algorithms); and service-chain operations (triage, escalation/rapid response, transfer, resource allocation, workflow, timeliness/delay, and handoffs). PubMed yielded 722 records and Scopus yielded 83 (total 805); and EndNote deduplication (Find Duplicates with manual verification) left 779 unique records. Titles/abstracts were screened for workflow-deployable, auditable evidence relevant to obstetric emergency pathways (timestampable trigger/ack/action steps, escalation/transfer completion, resource-ready milestones, override/closed-loop documentation, or missing data signals). We included 48 sources (peer-reviewed studies, guidelines, and implementation toolkits/standards, including web resources). Evidence was synthesized narratively (no meta-analysis). Exact search strings, limits, run date, and yields are provided in Supplementary Appendix S1. Supplementary Table S1 maps key MFAS components to representative supporting evidence and established anchors for quality improvement and clinical decision-support evaluation.

## 3 Current progress: standardization bundles, early warning, and the missing equity layer

### 3.1 PPH: from “quantification” to treatment bundles and updated toolchains

PPH care has evolved from subjective estimation toward quantified assessment that is explicitly linked to predefined treatment bundles, aligning measurement, pathway activation, and early treatment within a single operational toolchain (2, 8, 9). World Health Organization guidance pairs structured blood-loss assessment with a treatment bundle, operationalizing a “quantify-then-treat” approach rather than reliance on variable, clinician-specific thresholds (8). This consolidated pathway logic further embeds diagnosis and treatment within higher-quality perinatal care, implying stocked supplies, defined roles, and standardized handoffs rather than *ad hoc* escalation (1). Contemporary guidance also underscores that timely access to non-surgical hemorrhage-control devices constitutes a pathway-critical node (10). In practice, bundle implementation commonly fails at toolchain interfaces—when quantification does not trigger action, or when activation cannot reliably mobilize resources. For equity-focused evaluation, audits should therefore treat device readiness, blood-product access, operating room availability, and staffing capacity as measurable nodes and not merely verify that a “bundle” exists in policy documents (2).

### 3.2 Obstetric sepsis: screen → confirm → escalate and toolkit-based systems

Work on obstetric sepsis has converged on a two-step process: screening for risk and then confirming with focused evaluation and initiating escalation pathways that bundle diagnostics, antibiotics, fluids, and higher-acuity clinical review (4, 11). Toolkits operationalize this approach by packaging criteria, checklists, reassessment intervals, and escalation prompts to improve reliability across handoffs and shifts (11). Screening thresholds remain operationally consequential: increasing sensitivity can amplify false-positive alerts and workload, whereas missed cases delay escalation and definitive treatment (12). Equity-relevant evaluation, therefore, requires linking triggers to treatment timing and escalation completion rather than validating the screening instrument in isolation (4).

### 3.3 The remaining gap: we standardized “what to do” but not “who gets it, when, and whether resources arrive”

These advances increasingly standardize “what to do,” yet many systems do not routinely audit who is triggered, who receives rapid response, and whether time-critical resources are actually made ready when escalation is signaled (2, 6, 7). Early warning scores can harmonize recognition and escalation pathways, but equity depends on the reliability of downstream response and resource readiness across units, shifts, and entry routes into care (13). The persisting gap is routine post-deployment measurement of stratified delays and denials at the point of care—an audit layer that renders failures visible, attributable, and correctable (6).

## 4 Reframing fairness: from model metrics to service outcomes

In this study, AI-supported resource allocation refers to workflow-embedded risk stratification or decision support—ranging from rules-based early warning scores to machine-learning risk scores and alerting/triage clinical decision support—that can trigger escalation and alter the timing, destination, or prioritization of time-critical resources in obstetric emergencies (e.g., rapid-response paging/escalation calls, blood product release, operating room [OR]/ICU mobilization, and interfacility transfer). We evaluate these tools only when they are deployed at the point of care and coupled to an explicit action pathway that generates auditable timestamps across the service chain (Trigger → Response → Resource-ready and Disposition → Outcome). We exclude standalone prediction studies without deployment and tools that do not initiate escalation or resource routing. Accordingly, the practical boundary in this article is not whether the trigger is “AI” in a strict sense, but whether it is workflow-embedded and capable of changing escalation, prioritization, or resource routing in a way that is auditable across the chain of care.

System taxonomy and MFAS applicability. Although we use “AI-supported resource allocation” as an umbrella term, auditability and failure modes differ materially across three deployed system types: (A) static rules-based scores/threshold triggers (including early warning scores embedded in electronic health record [EHR] rules engines); (B) machine-learning risk scores that output a probability or risk tier and may be periodically updated; and (C) routing/queuing/priority engines that explicitly allocate scarce operational capacity (e.g., bed assignment, transfer prioritization, and operating room [OR] queueing). MFAS has a tool-agnostic core that applies to all three types (timestamps across the trigger → response → resource-ready → disposition chain, stratified delay distributions, documented exceptions, and a corrective-action register) but requires additional, type-specific audit artifacts: (i) for type A, explicit rule/threshold specification and change logs; (ii) for type B, model versioning, input availability monitoring, output/override logs, and post-update re-audit; and (iii) for type C, auditable queue state, prioritization rules, and counterfactual review of “who was deprioritized” under surge conditions. Table 1 operationalizes the tool-agnostic MFAS core as chain-of-care indicators, whereas the minimum type-specific auditable artifacts needed to make those indicators trustworthy (e.g., rule/model/routing change logs) are summarized in Supplementary Box S1.

**Table 1:** MFAS minimum indicator set: metrics, definitions, owners, and audit-to-action triggers.

Chain link (block)	Minimum indicator	Operational definition (timestamp anchor)	Primary data-field anchor (examples)	Accountable owner	Audit-to-action trigger
Trigger	Trigger rate + T1 completeness	Triggers per eligible presentations; % eligible encounters with valid, linkable T1 (earliest-of rule).	Alert issued/displayed; call activation; pathway start log; denominator from triage/ADT.	OB/ED governance + Health IT/QI	Drift or stratified gaps; rising missingness/linkage failures; E6 increase.
Trigger (QA)	Missed-trigger rate (sentinel + sample)	Among sentinel high-acuity events (plus fixed random sample), proportion without prior T1; brief E-code adjudication.	Sentinel registry (e.g., emergency OR, MTP, unplanned ICU transfer, and near-miss).	Quality/Safety	Recurrent missed triggers in a stratum; E0/E6 concentration.
Response	Trigger → qualified assessment	Time T1 → T2 (median/IQR; P90), using one site-standard T2 marker.	Team arrival log; structured “assessment started”; first bedside assessment timestamp.	OB/ED clinical lead	Widening stratified gaps; P90 drift; recurrent E4/E5.
Response	Trigger → pathway activation (T3, recommended)	Time T1 → T3; if T3 not captured, report missingness explicitly.	Orderset/protocol start; bundle activation; cart request; MTP activation.	OB/ED clinical lead + Health IT	Rising T3 missingness; divergence vs. T1 → T2; post-change discontinuity (re-audit).
Response (diagnostic quality)	Acknowledged, no action	% alerts acknowledged without T2 or T3 within the response window.	Alert ack logs + EHR action/assessment timestamps.	Quality/Safety + Health IT	Elevated overall/stratified rate; shift clustering; recurrent E4/E5.
Resource-ready and disposition	Resource-ready performance (resource-specific)	For each resource stream, report time T1 → T4 (resource) (median/IQR; P90) and % within target window; over-window intervals receive one E-code.	Blood issued/at-bedside; OR/anesthesia readiness; ICU/monitored bed assignment; transfer acceptance/transport readiness.	Resource owners (blood bank; OR/anesthesia; ICU/bed mgmt; transfer center)	Recurrent E3 (capacity); override spikes; widening stratified gaps; sustained P90 deterioration; recurrent E0/E4/E5 triggers corrective action.
Outcome	Near-miss/severe morbidity; in-hospital death + harm proxies	Outcomes anchored to T1 (local definition versioned); harm proxies include deterioration while waiting and unplanned ICU admission after incomplete escalation.	Registry + ADT/ICU transfer + procedure/death timestamps.	Quality/Safety	Widening stratified outcome gaps; discordance (better process metrics but worse outcomes).

1. Operational timestamp definitions (T1–T5) are provided in Box 2; Supplementary Table S2 provides the full implementation template and field-level variants. This table summarizes the minimum indicator set, ownership, and audit-to-action triggers and does not redefine timestamps. 2. Medians with IQR and P90 for time-to-event metrics are reported; time distributions (not pass/fail only) to support equity auditing across strata were displayed. 3. T3 is recommended (not required): sites unable to capture T3 should report T3 missingness explicitly rather than substituting weak proxies. 4. Resource-specific T4: Blood/OR/ICU/transfer readiness should not be collapsed into a single composite time; each resource stream should be reported separately. 5. Over-window intervals receive one primary E-code (Supplementary Box S2). By default, E1–E2 are treated as non-avoidable; recurrent E0/E4/E5 patterns trigger a time-bound corrective action entry (owner, lever, deadline, verification metric), while E6 triggers data/IT remediation to restore auditability. 6. Any change to trigger logic, thresholds, routing, interfaces, or data-field mappings requires versioned change control and re-audit of chain metrics and stratified gaps. Conceptual basis and governance principles are consistent with prior work on auditing deployed AI and operational equity (e.g., (13, 14)).

ADT, admission–discharge–transfer; E-code, exception code for over-window intervals; EHR, electronic health record; ICU, intensive care unit; IQR, interquartile range; MFAS, Minimum Fairness Audit Set; MTP, massive transfusion protocol; OR, operating room; P90, 90th percentile; QI, quality improvement; SLA, service-level agreement.

To make this distinction explicit for readers working in mixed-technology environments, Box 1 summarizes which MFAS elements are tool-agnostic (applicable to both rules-based and AI/ML-based triggers) vs. which audit artifacts are system-specific.

**Box 1 Distinguishing MFAS’s tool-agnostic core from system-specific audit artifacts**

MFAS tool-agnostic core (applies to any deployed trigger mechanism):

(1)auditable chain timestamps (T1–T5) from Trigger → Response → Resource-ready/Disposition → Outcome;

(2)stratified delay distributions (medians/IQR/P90) and “acknowledged-without-action” rates;

(3)documented exceptions/primary reason codes for over-window intervals; and

(4)an audit-to-action register binding each gap to an owner, a controllable lever, and a verification metric.

Additional system-specific artifacts (needed to keep the core auditable in mixed-technology settings):

**Type A (static rules/threshold triggers)**: explicit specification of rules/thresholds, routing logic, and versioned change logs (who changed what, when, and why).

**Type B (machine-learning risk scores)**: model versioning, input availability/drift monitoring, immutable output and override logs, and mandated re-audit after updates or threshold changes.

**Type C (routing/queuing/priority engines)**: auditable records of queue state and prioritization rules plus counterfactual review of who was deprioritized under surge/capacity constraints.

### 4.1 Model-layer vs. service-layer: why AUC/calibration cannot answer fairness

Model-layer metrics (AUC, calibration) quantify discrimination and fit within a dataset; they do not test whether deployment reliably translates risk signals into timely escalation and resource readiness at the bedside (7, 14). In obstetric emergencies, similar underlying clinical risks can enter the system through heterogeneous data pipelines and operational contexts—missing inputs, delayed documentation, variable monitoring intensity, and site- or shift-specific data streams—thereby changing when (or whether) an alert is generated, displayed, and owned (15, 16). Even when a trigger is produced, downstream performance is shaped by access frictions and operational constraints: language discordance, payer rules, and referral pathways can influence senior review and transfer acceptance, while blood bank, OR, and ICU capacity determine whether activation yields resource-ready timeframes (6). Fairness should therefore be assessed at the service layer after deployment by auditing the chain of care—Trigger → Response → Resource-ready/arrival → Disposition → Outcomes—so that disparities can be localized to a specific link and paired with actionable workflow, communication, or capacity levers rather than inferred from model performance alone (6, 14).

**Figure 1:**
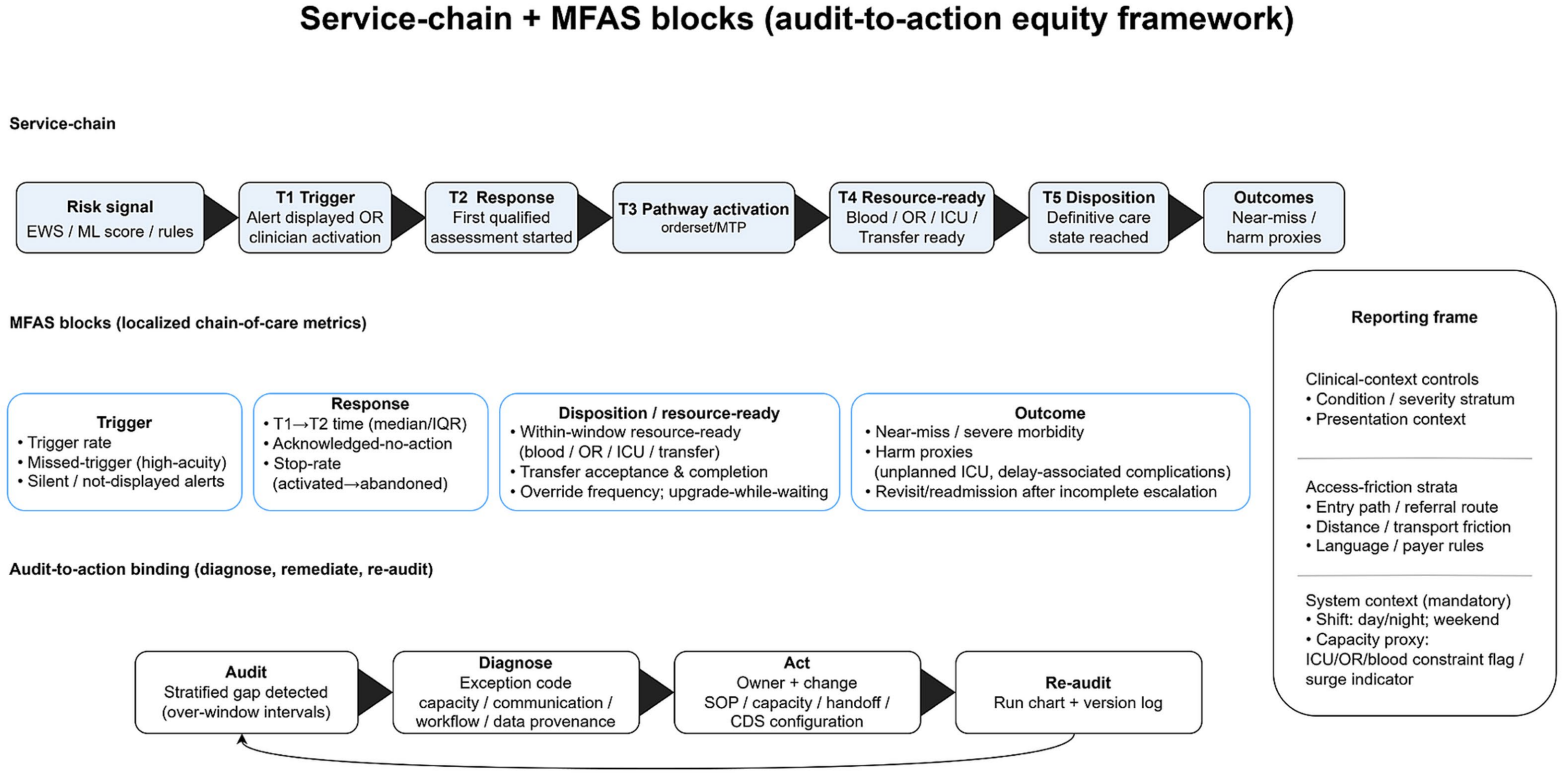
Service-chain + MFAS blocks (audit-to-action equity framework). The top row maps the service chain from risk signal (EWS/ML score/rules) to T1 trigger, T2 response, T3 pathway activation (order set/MTP), T4 resource-ready (blood/OR/ICU/transfer), T5 disposition (definitive care state reached), and downstream outcomes. The middle row specifies localized MFAS metrics across trigger, response, disposition/resource readiness, and outcome domains. The bottom row operationalizes “audit-to-action binding” (audit → diagnose → act → re-audit) to ensure detected stratified gaps drive accountable remediation and versioned re-evaluation. The right panel provides the reporting frame (clinical context controls, access-friction strata, and mandatory system context). AI, artificial intelligence; CDS, clinical decision support; EWS, early warning scores; ICU, intensive care unit; IQR, interquartile range; MFAS, Minimum Fairness Audit Set; ML, machine learning; MTP, massive transfusion protocol; OR, operating room; SOP, standard operating procedure; T1–T5, time-stamped care-chain milestones (trigger, response, pathway activation, resource-ready, and disposition).

Figure 1 summarizes MFAS as an audit-to-action instrument linking stratified delay gaps to remediable workflow, communication, and capacity levers.

### 4.2 Equity ≠ Equality: use avoidable delay as a shared denominator

Equality applies identical thresholds; equity aims to ensure comparable opportunities to receive time-critical care when risk is similar at presentation (7, 14). In obstetric emergencies, the most operational equity target is to reduce stratified gaps in avoidable delay, because delay is the mechanism through which triage, escalation, and resource contention translate into harm (17). We define avoidable delay as any chain-of-care interval (from trigger to key actions/resources) that exceeds a prespecified target window within the same clinical-context stratum, without a documented clinical contraindication or a patient-driven constraint. Target windows should be defined using a prespecified hierarchial approach: (1) existing local, executable service-level agreements when available; (2) guideline- or consensus-based time anchors translated into operational thresholds; and (3) when neither provides a usable anchor, a provisional window derived from baseline operational performance (e.g., pre-deployment P75/P90), with updates versioned and governed under MFAS change control. Each over-window interval receives one primary auditable E-code (Supplementary Box S2) during routine MFAS huddles; E1–E2 are treated as non-avoidable by default, recurrent E0/E4/E5 patterns trigger a time-bound corrective action entry (owner, lever, deadline, verification metric), and E6 is tracked as a data/information technology quality event requiring remediation to restore auditability. We operationalize delay using shared anchors mapped to the chain: trigger-to-activation, trigger-to-first qualified assessment, trigger-to-resource-ready (blood/OR/ICU/transfer readiness), and referral-completion delay, where applicable (13).

Target-setting guardrails to avoid institutionalizing inequity and to enable revision without obscuring gaps. When local baseline performance is used (e.g., pre-deployment P75/P90), it must be treated as a feasibility and capacity-planning input—not as a normative equity benchmark—because baseline workflows may already embed structural inequities. We therefore recommend a two-tier target structure reported in parallel for each interval: (i) an aspirational clinical anchor (derived from guideline intent, harm physiology, or cross-site best achievable performance, even if approximate), and (ii) a current capability target used for operational ramp-up. Critically, the current capability target must not be set solely by “what the baseline already delivers”; instead, it should be tied to a time-bounded improvement plan with explicit owners, controllable levers, and a prespecified ratchet schedule toward the clinical anchor (e.g., quarterly tightening). To prevent target revision from masking persistent inequities, routine reporting should (a) show side-by-side performance against both targets and (b) display absolute gap trajectories between strata (minutes/percentage points), not only whether each stratum “meets” a threshold. Sites should not systematically set looser targets for specific groups unless there is an ethical, clinically justified, and auditable rationale that is documented and subject to review. Any target modification must be versioned and accompanied by a re-audit to test whether gaps narrow without externalizing risk to safety or workload. *Illustrative example (PPH):* A site may define a trigger-to-activation clinical anchor of P90 ≤ 30 min, while adopting an interim capability target of P90 ≤ 45 min for the next 90 days during ramp-up, coupled to a ratcheting plan (e.g., ≤40 min next quarter and ≤35 min the quarter after) and named levers (e.g., timer ownership, auto-paging rules, and blood/OR readiness workflows).

### 4.3 Typical mechanisms of inequity in obstetric emergencies

Mechanisms should be treated as auditable. Data availability gaps arise when monitoring, documentation, or access to prior records differ across sites, shifting who triggers and when (15, 16). Workflow and capacity constraints—night shifts, crowding, and blood bank or operating room bottlenecks—can convert equal protocols into unequal waits (13, 17). Communication and advocacy gaps, including language discordance and limited social support, can shape escalation persistence and referral completion even when physiology is similar (6). AI can amplify these seams unless governance explicitly treats them as deployment targets (16).

## 5 The MFAS: a chain-of-care dashboard that forces actionability

### 5.1 Stratification: five minimum dimensions to separate clinical context from access frictions

To keep equity auditing feasible while preserving comparability, we recommend a small, auditable stratification set comprising (1) pregnancy phase (antenatal, intrapartum, and postpartum) (18); (2) baseline acuity at trigger (a low-burden severity marker captured at or immediately after T1) (19); (3) entry pathway (direct presentation, in-hospital deterioration, and interfacility transfer) (20); (4) geography/transfer proxy (catchment category or transfer-distance band; rurality where available) (21); and (5) access-to-care and communication-access proxies mapped to locally auditable fields—where available, payer/authorization status or self-pay as an administrative access proxy (22); and such data are not available or not meaningful, referral acceptance/transfer coordination steps, documentation completeness, rurality/travel-time bands, or other locally auditable administrative barriers, together with preferred language and interpreter need as communication-access indicators (23).

#### 5.1.1 Guardrails

MFAS uses workflow-facing, auditable strata to avoid converting equity measurement into identity profiling and treats data provenance and completeness as first-class audit targets because documentation gaps can shift who triggers and who receives action (14, 15).

#### 5.1.2 Optional but recommended protected-attribute stratification

The five “minimum” strata are designed to be low-burden and auditable within routine EHR workflows, but they are not a substitute for equity audits across protected characteristics when collection is lawful and governance permits are in place. Operational proxies (e.g., payer/authorization signals, referral/transfer pathway, and preferred language or interpreter need) are included only as auditable correlates of access friction; they should be treated as screening signals, not as substitutes for protected attributes, and must not be used to infer protected characteristics or to justify differential standards of care. We therefore recommend a second-tier stratification set—race/ethnicity, disability status, and other locally protected attributes—implemented under strict privacy and governance safeguards (role-based access, minimum cell-size rules, suppression of small counts, and community/ethics oversight where applicable). Where protected-attribute fields are unavailable, incomplete, or restricted, MFAS should explicitly report this limitation and avoid implying that proxy variables (e.g., payer status or language) fully capture structural inequities.

### 5.2 Four chain-of-care metric blocks: trigger → response → resource-ready and disposition → outcome

MFAS operationalizes equity as chain-of-care performance rather than model accuracy alone (13). Timestamp anchors and operational definitions (T1–T5) are provided in Box 2 (field-level EHR variants in Supplementary Table S2).

#### 5.2.1 Terminology

In this study, a trigger refers to the first actionable risk signal that is issued and owned by a named team (T1), pathway activation refers to the initiation of an executable order set/bundle (T3), and escalation is reserved as an umbrella term for step-up actions across the chain (e.g., senior review, paging cascades, transfer acceptance, and resource mobilization) rather than as a synonym for “trigger” or “activation.”

**Box 2 MFAS minimum data dictionary (deployment-ready)**

Timestamp anchors (T1–T5). Each encounter must be linkable by a unique encounter identifier, and all timestamps must be traceable to a primary source field (“system of record”).

T1 (Trigger issued/owned). T1 refers to the earliest time at which a trigger is generated and becomes actionable for a named team, e.g., an alert displayed in EHR, a rapid-response page sent, or a protocol trigger logged. An “earliest-of” rule should be applied across eligible trigger channels with both trigger type and trigger channel recorded.

T2 (First qualified assessment started). T2 refers to a site-standard marker for the first qualified bedside assessment by a designated responder (e.g., obstetrics senior review and rapid-response clinician). Each site should select one operational marker per site (team arrival log OR structured “assessment started”) and apply it consistently across time.

T3 (Pathway activation / first actionable order). T3 refers to the time an executable pathway is initiated (for example, a standardized order set is launched, a bundle is activated, a cart is requested, or a massive transfusion protocol is requested). If T3 is not reliably captured, its missingness should be reported explicitly, where weak proxies should not be substituted.

T4 (resource-ready, resource-specific). Separate readiness timestamps should be recorded for each critical resource stream; these should not be collapsed into a single composite:

∙T4-blood: blood products are bedside-ready (issued + delivered/available at bedside).

∙T4-OR: OR access confirmed AND anesthesia start time (or “patient in room” + anesthesia start, per site).

∙T4-ICU/monitored bed: monitored-bed assignment time (bed accepted for approval) and arrival time (if different).

∙T4-transfer: receiving acceptance time and transport-ready time.

∙T5 (Definitive disposition/arrival of definitive care). The time at which the patient reaches the definitive care state for the pathway (e.g., incision time for surgical hemorrhage control, ICU arrival, and definitive transfer arrival). This should be defined per pathway and per version of the definition.

Top-level exception codes (one primary E-code per over-window interval).

E0: Unknown/insufficient documentation (unattributed delay).

E1: Patient-driven constraint (declined/delayed consent and patient preference).

E2: Clinical contraindication/appropriate defer (documented medical reason).

E3: Capacity constraint (resource unavailable: blood/OR/ICU/transport).

E4: Handoff/communication failure (paging cascade, unclear ownership, and closed-loop failure).

E5: Process/policy friction (authorization/administrative barriers, routing rules, and transport workflow).

E6: Data/information technology failure (missing timestamps, alert downtime, and interface failure impairing auditability).

#### 5.2.2 Trigger

A fixed monthly random sample with brief reason-code adjudication using standardized exception codes (Supplementary Box S2) is used to measure the trigger rate and missed-trigger rate among high-acuity events. These events are adjudicated through sentinel-event review (near-miss/severe morbidity, unplanned ICU transfer, emergency OR, massive transfusion) (14).

#### 5.2.3 Response

The time from trigger to pathway activation (order sets, carts, and bundles), the time from trigger to first qualified bedside assessment, and the proportion of alerts acknowledged without subsequent action (13) were measured.

#### 5.2.4 Resource readiness and disposition

The proportion receiving key resources within target windows—blood products bedside-ready, operating room OR access/anesthesia start, monitored bed/ICU assignment, senior consultation—and, when applicable, transfer acceptance and completion (13, 17), were measured.

In MFAS, “resource-ready” is defined per resource stream using one locally auditable anchor (not an interchangeable event). For blood products, “bedside-ready” should indicate physical availability at the bedside (not merely released). For OR/ICU/transfer, the chosen anchor is explicitly specified (e.g., anesthesia start vs. wheels-in; staffed bed assignment vs. order placed; acceptance confirmed vs. transfer completed) and its data source is documented.

#### 5.2.5 Outcome

Near-miss/severe morbidity and in-hospital mortality were monitored, with additional evaluation of process-linked harm proxies such as clinical deterioration or care escalation while waiting and unplanned ICU admission after incomplete escalation (17). Table 1 maps the MFAS minimum indicator set to chain links, operational definitions (anchored to T1–T5), example data fields, accountable owners, and prespecified audit-to-action triggers (13, 14). These indicators are deliberately technology-agnostic and can be applied to rules-based early warning systems, along with AI/ML-based tools, provided the trigger is coupled to an executable pathway with auditable timestamps; system-specific governance artifacts are summarized in Supplementary Box S1.

Medians (with interquartile ranges) and proportions are reported, and time-to-event distributions are presented rather than relying solely on pass/fail indicators. This makes the equity question auditable: for whom did deployment translate prediction into timely resources and safer disposition (14)?

Table 1 should be used as an implementation checklist: (i) select the minimum indicators for the pathway and confirm local T1–T5 anchors (definitions in Supplementary Table S2); (ii) map each indicator to one auditable data source and assign an accountable owner; (iii) set target windows and stratifiers; and (iv) apply audit-to-action triggers to open a time-bound corrective action entry when gaps widen.

### 5.3 Audit-to-action binding: every metric must map to a controllable lever

MFAS affects equity only when each metric is explicitly bound to a controllable lever and a named owner (6, 7). If a response is delayed, timer ownership, auto-paging rules, and mandatory senior-review triggers should be defined. If resources do not arrive within target windows, cross-service prioritization rules across the blood bank, OR, and ICU should be implemented and published, and all log overrides (including who acted and why) should be recorded so that rationing is reviewable. If transfers fail, closed-loop transfer tracking with escalation should be deployed when no accepting bed is secured within the defined window, without delaying bedside stabilization and reassessment. Examples of lever-to-metric mapping are provided in Supplementary Box S3.

### 5.4 Display rules: show gaps as absolute difference + relative ratio + pre/post trends

Gaps should be displayed as absolute differences (minutes; percentage points), relative ratios, and pre/post run charts or statistical process control charts linked to specific workflow changes (24). *p*-values should be avoided as the headline metric; interpretability and actionability should be prioritized. Denominators should be kept stable and configuration changes should be annotated. Versioning and change control should be applied to model inputs, thresholds, alert routing, and workflow rules because configuration shifts can redistribute delays and resources in practice (14). Equity drift should be treated as a patient-safety risk: a baseline should be established before go-live, continuous monitoring should be conducted after launch, and the tool should be downgraded, rolled back, or disabled if harms or widening gaps emerge (6).

Minimum governance includes role-based access; immutable logs for model outputs, overrides, and pathway activations; and auditable system interfaces aligned with responsible-AI guidance and risk-management expectations. A minimum auditable artifact set is summarized in Supplementary Box S1 (16, 25). To ensure measurement low-burden and comparable across sites, a minimal, implementation-ready data-capture template (including required fields and timestamp definitions) is provided in Supplementary Appendix S2.

## 6 Case application: equitable allocation in PPH, hypertensive crises, and obstetric sepsis

### 6.1 Three pathway “resource bottleneck maps”

For PPH, bottlenecks tend to cluster around blood products (ordering, release, and transport), hemorrhage-control devices, rapid access to the OR or interventional radiology, anesthesia availability, and activation of massive transfusion pathways (1, 8, 10). Hypertensive crises/eclampsia require timely administration of magnesium sulfate and intravenous antihypertensives with continuous monitoring, supported by monitored-bed capacity and rapid escalation to higher-acuity monitoring or transfer when neurologic risk escalates (3, 13). Obstetric sepsis depends on a reliable screen-to-confirm sequence, prompt culture collection and laboratory testing, timely antimicrobials and resuscitation, and escalation pathways; ICU capacity and transfer logistics remain recurrent constraints (4, 11).

### 6.2 Translating equity questions into MFAS metrics

#### 6.2.1 PPH

Within comparable severity strata, it should be assessed whether referred arrivals experience later pathway activation (T3) by tracking trigger-to-activation time, the acknowledged-without-action proportion, and time to blood (or device) bedside readiness, stratified by entry pathway and transfer-distance proxies (8, 10).

#### 6.2.2 Hypertensive crises

Trigger-to–magnesium sulfate initiation, trigger-to–intravenous antihypertensive initiation, time to monitored-bed arrival, and transfer completion (when applicable) should be tracked, stratified by entry pathway and distance/transport proxies (3, 13).

#### 6.2.3 Sepsis

It should be evaluated whether communication barriers or incomplete closed-loop steps delay reassessment and escalation by tracking trigger-to-reassessment time, escalation completion, and missing-data rates for key vital signs and laboratory results, stratified by interpreter need (or another auditable communication proxy) and co-reported with shift and capacity context (4, 11). Box 3 provides a synthetic worked example illustrating how these MFAS metrics are summarized (median/ interquartile range [IQR] and P90 and gap measures), adjudicated using E-codes, and translated into an owned audit-to-action entry.

### 6.3 Worked example: synthetic MFAS dashboard, adjudication, and audit-to-action

**Box 3 Worked example: synthetic MFAS dashboard (PPH)**

∙Cohort (synthetic): 16 PPH alerts meeting eligibility; strata = referred transfer (n = 8) vs. direct arrival (n = 8). Severity controlled via shock index band at T1.

∙Chain metrics (minutes): T1 → T3 (trigger → pathway activation): Direct 6 (IQR 4–10), P90 18; Transfer 14 (IQR 9–22), P90 42. Gap (P90): +24 min.

∙Chain metrics (minutes): T1 → T4 (trigger → blood bedside-ready): Direct 22 (IQR 16–30), P90 55; Transfer 38 (IQR 26–60), P90 120. Gap (P90): +65 min.

∙Acknowledged-but-no-action: Direct 1/8 (12.5%); Transfer 3/8 (37.5%).

∙E-code adjudication (examples): Case T-03: T1 logged, T3 delayed; blood released on time but transport delay documented → E3 (capacity constraint: logistics bottleneck). Case T-07: alert acknowledged, no pathway activation until clinical deterioration; no override reason documented → E0 (unknown/insufficient documentation; unattributed delay).

∙Audit-to-action entry: Owner: Blood bank + transport lead (responsible); OB hemorrhage QI (accountable). Lever: pre-authorization for transfer PPH alerts; “blood-to-bedside” courier escalation rule; enforce override-reason field. Deadline: 30 days. Verification: the next-month run chart shows Transfer T1 → T4 P90 ≤ 80 min; E0 rate <10%; balancing measure = false activations + staff workload unchanged.

This worked example demonstrates how stratified delay gaps are interpreted with explicit exception coding and converted into accountable corrective actions and balancing measures, as discussed in the next section.

## 7 Discussion

### 7.1 Equity–safety tension and how MFAS avoids performative fairness

A predictable concern is that lowering thresholds or broadening triggers in the name of equity could increase false alarms, consume scarce OR/ICU/blood capacity, and worsen overall outcomes through crowding and mis-prioritization (26–28). MFAS treats this as a design constraint rather than a trade-off to be dismissed. First, MFAS audits equity on chain performance conditional on comparable presenting risk, not on raw trigger counts; any trigger expansion must therefore be paired with explicit resource-prioritization rules and surge plans (29). Second, MFAS binds stratified gaps to actionable levers: when delays shift from missed triggers to capacity constraints, the appropriate response is capacity governance (E3), rather than progressively expanding alerting thresholds (29). Third, MFAS prevents performative reporting by requiring versioned change control, override review, and a corrective-action register with named owners, deadlines, and verification metrics (30). Equity, therefore, becomes routine quality control—reducing avoidable delay without externalizing risk to safety, workload, or other patients.

### 7.2 Governance: accountability, transparency, and risk management for equity

MFAS governance requires cross-department ownership—obstetrics, anesthesia, ICU, blood bank, quality/safety, and health information technology (with data science support as needed)—to prevent an accountability vacuum in which a vendor “owns” the algorithm while clinicians own the consequences (6). A minimal cadence is sufficient: a monthly MFAS dashboard huddle reviews stratified run charts by chain link and assigns owners for clusters of over-window intervals; a quarterly cross-service review reassesses target windows, routing rules, and capacity plans; and sentinel events trigger rapid reconstruction of timestamps and immediate containment actions (14).

Transparency should be practical. Patients and families should receive a plain-language written explanation that the system is designed to support timely response—not to deny care—with a correction/appeal pathway when data are missing or an escalation decision is disputed (6, 16).

*Patient-facing example text*: “We use an electronic alert to help the care team respond quickly when time-critical pregnancy or postpartum complications are suspected. The alert supports timely assessment and escalation and does not replace clinician judgment or deny care. If you believe key information was missing or escalation was delayed, please ask the bedside team to review the alert record and escalation steps.” *Correction/appeal pathway*: (1) a bedside review of the alert and timeline; (2) submission of a data-correction ticket within 24–48 h if documentation/logs are incomplete; (3) a service-lead review of MFAS timestamps/E-code within 7 days if escalation is disputed; (4) document actions in the audit-to-action register and provide a brief written summary to the patient/family. Internally, transparency requires acknowledgment and override logs so that “who acted and why” remains visible for post-event learning and accountability (16).

#### 7.2.1 Portability across health systems and resource contexts

MFAS is designed as a system-agnostic audit logic: the universal elements are (i) the chain-of-care timestamp anchors (T1–T5); (ii) a single primary exception/adjudication code per over-window interval; (iii) an audit-to-action register with named owners, deadlines, and verification metrics; and (iv) balancing measures (workload, false activations, downstream delays). What varies across settings is the operational definition and data anchor for “resource-ready” (e.g., blood at bedside vs. released from blood bank; OR “wheels-in” vs. anesthesia start; monitored-bed assignment vs. arrival) and the feasibility of specific stratifiers. We use WHO guidance to anchor clinically expected trigger-to-treatment transitions where applicable; operational toolkits (e.g., regional bundles) are cited as implementation examples rather than jurisdictional requirements. Where payer/authorization fields are not collected or not meaningful, sites can substitute auditable administrative/access proxies such as referral source and acceptance steps, transfer coordination timestamps, documentation completeness, rurality/travel-time bands, language/interpreter need, and shift/capacity context. For cross-site interpretability, each site should explicitly document, for each resource stream, the chosen operational anchor and its data source.

#### 7.2.2 Balancing measures

MFAS-triggered “equity fixes” should not increase net harm by overloading teams, crowding critical resources, or delaying other emergencies. Therefore, any corrective action plan should be paired with balancing measures monitored on the same cadence as MFAS gaps: (i) alert burden and quality (volume, acknowledgment latency, false activations), (ii) workforce load (task burden, overtime, staffing ratios where available), (iii) critical-capacity spillover (OR/anesthesia utilization, ICU/monitored-bed occupancy/boarding), and (iv) non-index pathway impact (downstream delays for other emergencies). If balancing measures worsen beyond a predefined threshold, the action is throttled, redesigned, or rolled back.

### 7.3 Future directions

#### 7.3.1 Research directions

Research should test whether MFAS chain metrics (trigger → response → resource-ready/arrival → disposition/outcome) mediate changes in maternal outcomes using pragmatic designs, including quasi-experiments, stepped-wedge rollouts, and quality improvement evaluations with interrupted time series. Capacity constraints (beds, blood products, and OR access) should be modeled as exposures and moderators and measured contemporaneously rather than treated as *post hoc* explanations. Complement quantitative audits with brief qualitative huddles to attribute failures to specific chain links. Post-deployment equity evaluation remains essential because workflow frictions determine who benefits or is harmed—effects that offline AUC and calibration cannot reveal (14). To make MFAS empirically testable, future studies can pair these designs with pre-specified MFAS endpoints (e.g., T1–T5 interval distributions, P90 avoidable delay, and disparity-in-delay gaps) and implementation measures (trigger acknowledgment, override rates, escalation adherence, and capacity constraints). Where randomization is infeasible, difference-in-differences using matched comparison units/sites can strengthen causal attribution by separating MFAS-related workflow change from secular trends.

#### 7.3.2 Policy and practice directions

Policy should embed MFAS within maternal quality accountability and regional referral-network governance to enable routine comparability across institutions rather than isolated local dashboards (1). This approach aligns with World Health Organization pathway logic: what should occur in obstetric emergencies should be standardized and routine audits should be conducted on who receives it and when (1). Operationalization should adopt risk-management language—treating equity as an accountable, measured, managed risk item—consistent with the National Institute of Standards and Technology AI Risk Management Framework (25).

More broadly, MFAS aligns with emerging HealthCare/Medicine 5.0 thinking that treats digital innovation as a human-centered, resilient socio-technical system; under this lens, avoidable delay is not only an equity-relevant operations KPI but also a marker of system adaptability under stress (31).

Recent system-ethics work cautions against reducing fairness to isolated subgroup metrics and emphasizes intersectionality and interdisciplinary accountability; MFAS complements this stance by auditing service-chain outcomes that integrate compounding structural frictions while still enabling protected-attribute analyses when lawful and governance permits (32).

As clinical AI moves toward more goal-directed, semi-autonomous (“agentic”) behaviors in triage and escalation, equity risks may shift from static prediction error to dynamic decision policies and cross-service propagation—further motivating pathway-level audits and auditable action traces/overrides (33).

Practically, MFAS can function as a post-deployment monitoring layer that complements recent consensus guidance on trustworthy, deployable medical AI and pragmatic operations work on ambient AI (34, 35).

## 8 Conclusion

If AI in obstetric emergencies does not reduce stratified gaps in avoidable delay, it should not be considered a successful digital public health intervention. The decisive test lies in deployment: whether a triggered pathway delivers timely assessment, resource-ready care, definitive disposition, and safer outcomes—and whether these gains are shared across shifts, sites, and entry pathways. The minimum viable route is practical and auditable: the MFAS dashboard (Figure 1) should be implemented, each metric should be assigned to a named owner and linked to a controllable lever, and an accountability chain that governs timers, prioritization rules, and override review across departments should be run. Equity should function as routine quality control—reported in standard dashboards, reviewed in post-event learning loops, and corrected through rapid, documented fixes—so that risk signals reliably translate into resources at the bedside rather than remaining a one-off publication claim.

## Data Availability

The original contributions presented in the study are included in the article/Supplementary material, further inquiries can be directed to the corresponding author.
