# Supplementary material for: Equity at the point of care: auditing AI-supported resource allocation in obstetric emergencies
Source: Front Public Health. 2026 Mar 3;14:1774367. doi: 10.3389/fpubh.2026.1774367 (PMC12992295; doi:10.3389/fpubh.2026.1774367)
Supplement: Supplementary file 1 [file Supplementary_file_1.zip › Supplementary Box S2.DOCX]

**Supplementary Box S2. Exception codes for over-window intervals (MFAS)**

Purpose: Convert “safety narratives” into auditable, comparable reasons for delay.

Coding rule: Assign exactly ONE primary code per over-window interval. Add ONE optional secondary code only when needed for learning (e.g., recurrent patterns). If “Other” is used, a short free-text specification is required.

| Primary exception codes (E-codes)  E0. No documented exception (default)  • Definition: The interval exceeded the target window and no exception was documented.  • Audit handling: Treated as potentially avoidable and prioritized for rapid review.  E1. Clinical contraindication / required stabilization (non-avoidable by default)  • Definition: Delay was clinically necessary to stabilize the patient or because proceeding would be unsafe.  • Examples: airway/breathing/circulation stabilization; ongoing resuscitation; urgent competing clinical emergency documented in record; required preconditions not met (e.g., anesthesia safety prerequisites).  E2. Patient-driven constraint (non-avoidable by default)  • Definition: Delay primarily driven by patient or surrogate decision-making constraints.  • Examples: refusal; delayed consent; family decision delay; patient preference necessitating additional counseling (documented).  E3. Capacity constraints  • Definition: A required resource was unavailable within the window due to system capacity limits.  • Examples: bed/OR/ICU/blood/transport unavailable; staffing limits/surge conditions; receiving-facility capacity denial.  E4. Communication / escalation breakdown  • Definition: Delay due to failed alert delivery, acknowledgement, escalation, or handoff.  • Examples: alert not displayed/routing failure; escalation not acknowledged; handoff breakdown (unit-to-unit or interfacility).  E5. Workflow / process breakdown  • Definition: Delay due to protocol/process execution failures within the care pathway.  • Examples: orderset/protocol not activated; task sequencing error (orders placed but not executed); missing equipment/cart readiness.  E6. Data provenance / measurement issues (data-quality event; not a clinical “excuse”)  • Definition: The interval cannot be reliably adjudicated due to missing/unreliable timestamps or record linkage issues.  • Examples: missing/unreliable timestamps (clock drift, delayed charting); mislinked encounters/duplicate records; documentation latency prevented real-time triggering.  • Audit handling: Tracked separately as a data/IT quality problem and triggers corrective action to restore auditability.  E7. Other — specified  • Definition: Reason does not fit E1–E6.  • Requirement: Must include ≤15 words free-text specification; otherwise re-coded as E0.  Secondary code (optional, for learning only)  • Use only when it materially helps pattern detection (e.g., “interfacility transfer” + “capacity denial”).  • The primary code remains the official auditable reason.  Adjudication note (audit-to-action binding)  • Code assignment should be completed during routine MFAS dashboard huddles and verified in sentinel-event rapid reviews.  • Recurrent E0/E4/E5 patterns should trigger a corrective action entry (owner, lever, deadline, verification metric).  • Goal: system learning and reliability improvement, not individual blame. |
| --- |

**Abbreviations:**E-code (E0–E7), exception code for over-window intervals; ICU, intensive care unit; IT, information technology; MFAS, Minimum Fairness Audit Set; OR, operating room.
