# Supplementary material for: Equity at the point of care: auditing AI-supported resource allocation in obstetric emergencies
Source: Front Public Health. 2026 Mar 3;14:1774367. doi: 10.3389/fpubh.2026.1774367 (PMC12992295; doi:10.3389/fpubh.2026.1774367)
Supplement: Supplementary file 1 [file Supplementary_file_1.zip › Supplementary Box S1.DOCX]

**Supplementary Box S1. Governance artifacts (minimum viable set)**

| 1) Monthly annotated run charts (by chain link and stratum)  • Cadence: monthly (plus ad hoc after sentinel events)  • Owner: Quality/Safety + OB governance (Responsible); service leads (Accountable)  • Minimum contents: median/IQR and P90 for each chain link (T1→T2, T1→assessment, T1→resource-ready, referral completion where applicable); stratified gaps (absolute + ratio); exception mix (E0–E7); major operational notes (surge, staffing, policy changes).  • Action triggers: sustained worsening for 2 consecutive periods; widening stratified gaps; spike in E0/E4/E5.  2) Corrective-action register (audit-to-action binding)  • Cadence: updated in real time; reviewed monthly  • Owner: interdisciplinary owner group chair (Accountable); named issue owner (Responsible)  • Minimum contents: issue statement; linked metric(s); hypothesized failure mode; lever(s); owner; deadline; verification metric + target; status; closure criteria.  • Action triggers: any recurrent E0/E4/E5 pattern; high-frequency E6; repeated capacity constraints (E3) without mitigation plan.  3) Configuration / version-change log (model + interface + routing)  • Cadence: every change (no exceptions)  • Owner: Health IT (Responsible); clinical governance (Accountable)  • Minimum contents: what changed (inputs/thresholds/routing/UI); rationale; expected impact; effective date/time; rollback plan; re-audit requirements; sign-off.  • Action triggers: any threshold/interface/use change requires re-audit of chain metrics and stratified gaps.  4) Override review summary (frequency, reasons, equity implications)  • Cadence: monthly (or weekly during go-live), plus sentinel-event review  • Owner: service leadership + Quality/Safety (Accountable); Health IT provides logs (Responsible)  • Minimum contents: override rate; top reasons; time-of-day/shift patterns; stratified distribution; downstream outcomes (e.g., resource-ready delays); actions taken.  • Action triggers: override rate above predefined ceiling; stratified override asymmetry; overrides associated with increased avoidable delay. |
| --- |

**Abbreviations:**
IQR, interquartile range; IT, information technology; MFAS, Minimum Fairness Audit Set; OB, obstetrics/obstetric; P90, 90th percentile; UI, user interface; T1, trigger timestamp; T2, response/assessment timestamp; E-code (E0–E7), exception code for over-window intervals.
