# Supplementary material for: Equity at the point of care: auditing AI-supported resource allocation in obstetric emergencies
Source: Front Public Health. 2026 Mar 3;14:1774367. doi: 10.3389/fpubh.2026.1774367 (PMC12992295; doi:10.3389/fpubh.2026.1774367)
Supplement: Supplementary file 1 [file Supplementary_file_1.zip › Supplementary Table S2.DOCX]

**Supplementary Table S2. Minimum viable timestamp dictionary for MFAS**

| **Timestamp** | **Minimum required** | **Operational definition (one sentence)** | **Primary data-field anchor (examples; pick one and standardize)** | **Edge cases / hierarchy rules** |
| --- | --- | --- | --- | --- |
| T1 — Trigger (time zero) | Yes | The earliest moment an escalation pathway is activated: first automated alert displayed to a responsible clinician/team OR first clinician-initiated escalation activation, whichever occurs earlier. | Alert log “displayed/issued”; rapid response / obstetric emergency call activation log; protocol/bundle activation log. | Earlier-of rule applies when both automated alert and clinician activation occur. If both exist, optionally store both components (alert time; clinician-activation time) but report T1 as the earlier. If alert is generated but never displayed, do not use it as T1 unless the system reliably indicates “presented to user.” |
| T2 — Response (first qualified bedside assessment) | Yes | The time a qualified responder arrives at bedside or initiates a documented real-time assessment, whichever is earlier and reliably captured. | Team arrival timestamp (if logged); first bedside note time; first “assessment started” entry in emergency pathway documentation. | If only note-time exists and is known to lag, document this limitation and keep it consistent. For remote triage (e.g., tele-consult), define response as first real-time assessment interaction documented (call start time) and apply consistently. |
| T3 — Pathway activation (orderset/protocol execution start) | No (recommended) | The time the formal pathway is operationalized (e.g., orderset opened/signed, cart requested, bundle activated, MTP activated). | Orderset signed time; protocol/bundle activation; MTP activation log; sepsis bundle initiation. | Use T3 to separate “assessment delay” from “execution delay.” If T3 is unavailable, omit and report “not captured” rather than substituting an imprecise proxy. |
| T4 — Resource-ready/arrival (primary operational endpoint) | Yes | The time the time-critical resource is available for use (ready at point of care or definitively secured) using a resource-specific “ready” marker that is pre-specified by the site. | Blood products: first unit issued/dispensed OR arrived on unit. OR/anesthesia: patient in-room OR anesthesia start (choose one). ICU: bed assigned + acceptance confirmed OR ICU arrival (choose one). Senior consult: consultant arrival time OR consult-start time. Transfer: acceptance confirmed (bed/attending acceptance), plus transport depart/arrive if tracked. | Do not mix “requested” with “ready” markers in reporting—keep request (T3) separate from ready (T4). If multiple resources are tracked, report each as its own T4-* subtype (e.g., T4-blood, T4-OR) with a shared definition template. If capacity constraints cause staggered readiness (e.g., blood available but OR delayed), report each resource stream separately rather than forcing one composite time. |
| T5 — Disposition (definitive care state reached) | Yes | The time the patient reaches the next definitive care state (e.g., arrival in OR/ICU/higher-acuity unit, or transfer departure/arrival) using a single site-standard disposition marker. | Location/ADT transfer time; OR in-room time (if used as disposition); ICU arrival time; transfer departure time (or arrival time, if that is the site standard). | Pre-specify one disposition marker for reporting; optionally retain additional markers (e.g., transfer depart and arrive) for internal ops but avoid switching midstream. If disposition is canceled/reversed, record cancellation reason code but keep the original timestamps auditable. |
| T6 — Closed-loop completion / safety-step completion | No (scenario-specific) | The time a pathway-defined safety step is completed (e.g., documented handoff complete, scheduled follow-up completed when explicitly included as a safety step). | Handoff completion checkbox/time; follow-up appointment completion in scheduling system; discharge safety-call completion. | Use only when the pathway explicitly includes the step as a safety control; otherwise omit to avoid scope creep. Report completion rate and time-to-completion separately from acute chain metrics. |
| T7 — Outcome event time (for window anchoring) | No (outcomes required, timestamp optional) | The time an outcome endpoint occurs, anchored to T1 for time-window reporting (e.g., near-miss/severe morbidity event time, in-hospital death time, unplanned ICU transfer time, readmission/ED revisit time). | Event timestamp in clinical registry; ICU transfer time; procedure time; death time; readmission/ED arrival time. | Outcomes should be reported with pre-specified windows anchored to T1 (e.g., in-hospital; 72h; 30-day). If event timing is unreliable, report the outcome occurrence but flag event-time as “not captured.” |

**Table notes (recommended)**

1. **Clock and linkage:** All timestamps should use a single time standard (system time) and be linkable to a single encounter/episode ID; sites should monitor clock drift between the EHR, alerting/CDS system, blood bank, OR system, ICU bed-management/ADT feed, and transport logs.
2. **Consistency over completeness:** If a site cannot capture optional fields (e.g., T3/T6) reliably, MFAS remains implementable using **T1–T2–T4–T5**; report missingness explicitly rather than substituting weak proxies.
3. **Change control:** Any change to trigger logic, alert display behavior, pathway definitions, or timestamp anchors must be versioned and annotated on trend charts to prevent artificial “improvement” from definition changes.
4. **Resource-specific T4:** If multiple resources are audited (e.g., blood products and OR access), record them as distinct subtypes (e.g., T4-blood, T4-OR) using pre-specified “ready” markers, rather than collapsing into a single composite time.

**Abbreviations**

**ADT**, admission–discharge–transfer; **AI**, artificial intelligence; **CDS**, clinical decision support; **ED**, emergency department; **EHR**, electronic health record; **ICU**, intensive care unit; **MFAS**, Minimum Fairness Audit Set; **MTP**, massive transfusion protocol; **OR**, operating room.
