# Supplementary material for: Equity at the point of care: auditing AI-supported resource allocation in obstetric emergencies
Source: Front Public Health. 2026 Mar 3;14:1774367. doi: 10.3389/fpubh.2026.1774367 (PMC12992295; doi:10.3389/fpubh.2026.1774367)
Supplement: Supplementary file 1 [file Supplementary_file_1.zip › Supplementary Appendix S2.DOCX]

**Supplementary Appendix S2. Implementation-ready data capture template**

Core identifiers and stratification fields (de-identified):

- Encounter ID (de-identified); facility/site ID if multi-site.
- Pathway type: PPH / hypertensive crisis (eclampsia/severe HTN) / obstetric sepsis (or other specified).
- Pregnancy phase band (e.g., intrapartum, postpartum ≤24 h, postpartum >24 h).
- Entry pathway: direct vs referral; entry location (ED vs clinic vs inpatient).
- Baseline acuity marker at trigger (choose one per pathway; see menu below).
- Transfer proxy: transfer required (Y/N); distance band if available.
- Communication access proxy: preferred language recorded; interpreter needed (Y/N).
- System context: shift (day/evening/night) and one capacity proxy flag (e.g., surge staffing, bed saturation) where available.

Baseline acuity marker menu (examples; select locally auditable fields):

- PPH: shock index band and/or MTP triggered (Y/N).
- Hypertensive crisis: severe-range BP with neurologic symptoms (Y/N) and/or seizure (Y/N).
- Sepsis: lactate band and/or vasopressor within a prespecified window (Y/N) and/or organ support requirement (Y/N).

Timestamps (use NA when not applicable; record system time where possible):

For each timestamp capture: (i) event time, (ii) source_system (EHR/paging/transfer center/blood bank LIS/other), and (iii) source_field_name (local field or event code).

**T1 trigger:** Time of trigger event (clinical or algorithmic) initiating the pathway.

**T2 alert queued/displayed:** System time when the alert entered the intended recipient’s queue/inbox (not when later read).

**T3 acknowledgement:** System time of recipient acknowledgement (button/callback receipt/phone handback) using a standardized mapping.

**T4 pathway activation + resource request/assignment:** Time of pathway activation (orderset/protocol) and initial resource request or assignment (e.g., blood order, OR request, ICU bed request).

**T5 resource-ready and disposition executed:** Capture as two sub-timestamps when feasible: T5a resource-ready (blood issued/OR ready/ICU bed assigned/transport ready) and T5b disposition executed (procedure start/ICU arrival/transfer departure/arrival).

Over-window exception coding (per Supplementary Box S1):

- primary_E_code (E0–E7; exactly one per over-window interval).
- secondary_E_code (optional; for learning only).
- free_text_if_E7 (≤15 words; required if E7 selected).

Process flags:

- acknowledged-no-action (Y/N): acknowledgement present without downstream activation/action within the target window (define locally).
- pathway stop/abandon (Y/N; brief reason).
- override used (Y/N; override reason).

Outcomes (minimum):

- near-miss / severe morbidity indicator (local definition) + definition_reference (name/version/date).
- unplanned ICU transfer (Y/N).
- in-hospital death (Y/N).
- deterioration/upgrade while waiting (Y/N).

**Abbreviations:**
BP, blood pressure; ED, emergency department; EHR, electronic health record; HTN, hypertension; ICU, intensive care unit; LIS, laboratory information system; MTP, massive transfusion protocol; NA, not applicable; OR, operating room; PPH, postpartum hemorrhage; T1–T5, time-stamped care-chain milestones; Y/N, yes/no; E-code (E0–E7), exception code for over-window intervals.
