# Supplementary material for: Equity at the point of care: auditing AI-supported resource allocation in obstetric emergencies
Source: Front Public Health. 2026 Mar 3;14:1774367. doi: 10.3389/fpubh.2026.1774367 (PMC12992295; doi:10.3389/fpubh.2026.1774367)
Supplement: Supplementary file 1 [file Supplementary_file_1.zip › Supplementary Table S1.DOCX]

**Supplementary Table S1. Mapping key MFAS components to established QI/CDS evaluation anchors and supporting sources**

| **Key MFAS component** | **QI/CDS evaluation anchor (why this component is justified)** | **Representative supporting references (as cited in the manuscript)** |
| --- | --- | --- |
| Acknowledged-without-action (AWA) (alert acknowledged but no pathway activation/escalation) | CDS evaluation frameworks emphasize assessing responses and downstream actions, not only alert firing; AWA operationalizes “signal received but not acted upon.” | McCoy et al. framework for evaluating CDS alerts and responses [29]; inpatient CDS override/response patterns [27] |
| Override / acknowledgment logs (who acted/overrode, why, and what happened next) | Monitoring override behavior and documenting rationale/outcomes supports accountability and identifies workflow mismatch or unsafe alerting. | Inpatient alert overrides [27]; CDS alert malfunction taxonomy (failure modes that require monitoring) [30] |
| Balancing measures (alert volume, workload proxies, false activations, downstream delays) | Implementation evaluation requires tracking benefit–harm tradeoffs and preventing “harm-shifting” (e.g., alert fatigue, workload overload, spillover delays). | Alert fatigue and repeated alerts under workload/complexity [26]; inpatient alert overrides [27]; CDS malfunction taxonomy [30]; AI risk monitoring and controls (AI RMF) [25]; governance/monitoring guidance for AI in health [16] |
| Resource-ready indicators (blood products, OR/anesthesia start, monitored-bed arrival, transfer completion) | Time-critical obstetric care is pathway-based; guidelines/toolkits define clinically expected transitions, while MFAS operationalizes system readiness milestones as auditable events. | WHO PPH bundle guidance [8] (and consolidated WHO PPH guideline [1]); CMQCC hemorrhage toolkit [2]; ACOG update on hemorrhage-control devices (resource availability) [10]; Surviving Sepsis Campaign guideline [4]; CMQCC obstetric sepsis toolkit [11]; Levels of Maternal Care (escalation/transfer capability) [20] |
| Equity as a service outcome (stratified gaps in avoidable delay across chain links) | Equity assessment in clinical AI should be grounded in real-world workflow and outcome disparities, not model metrics alone. | Guiding principles on algorithm bias and disparities [6]; algorithmic fairness in AI for healthcare [7]; accuracy and equity viewpoint [14]; STANDING Together consensus recommendations [15] |
| Governance + change control / re-audit (any threshold/interface/routing change triggers re-audit) | Responsible AI/CDS requires continuous monitoring, versioning, and re-assessment after changes due to configuration/drift risk. | NIST AI Risk Management Framework (AI RMF 1.0) [25]; WHO ethics and governance guidance [16] |
| Run charts and audit cadence (dashboard review of stratified timeliness and gap metrics) | QI practice commonly uses time-series/run charts to visualize performance and support governance oversight. | Time-series display for hospital boards [24] |

**Note:** References are provided as **representative evidence anchors** linking each MFAS component to established QI/CDS evaluation logic and/or widely used implementation guidance; this mapping is not intended to be exhaustive.

Abbreviations: **MFAS**, Minimum Fairness Audit Set; **QI**, quality improvement; **CDS**, clinical decision support; **AI**, artificial intelligence; **PPH**, postpartum haemorrhage; **AWA**, acknowledged-without-action; **OR**, operating room; **RMF**, Risk Management Framework; **NIST**, National Institute of Standards and Technology; **WHO**, World Health Organization; **CMQCC**, California Maternal Quality Care Collaborative; **ACOG**, American College of Obstetricians and Gynecologists.
