# Supplementary material for: Equity at the point of care: auditing AI-supported resource allocation in obstetric emergencies
Source: Front Public Health. 2026 Mar 3;14:1774367. doi: 10.3389/fpubh.2026.1774367 (PMC12992295; doi:10.3389/fpubh.2026.1774367)
Supplement: Supplementary file 1 [file Supplementary_file_1.zip › Supplementary Appendix S1.docx]

**Appendix S1. Database search strings, limits, and run date**

**Run date (both databases):** 30 December 2025
**Language:** English
**Evidence window:** 1 January 2019 to 18 October 2025

**S1.1 PubMed (MEDLINE)**

**Database:** PubMed
**Date limits (PDAT):** 2019/01/01–2025/10/18
**Language filter:** english[LANG]
**Results (run date 30 Dec 2025):** 722 records

**Exact search string (copy/paste):**

( ("postpartum hemorrhage"[TIAB] OR "obstetric hemorrhage"[TIAB] OR eclampsia[TIAB] OR "hypertensive emergency"[TIAB] OR "hypertensive crisis"[TIAB] OR "maternal sepsis"[TIAB] OR "obstetric sepsis"[TIAB] OR "maternal early warning"[TIAB] OR "early warning score"[TIAB] OR "early warning system"[TIAB]) AND ("clinical decision support"[TIAB] OR CDS[TIAB] OR alert*[TIAB] OR "early warning"[TIAB] OR "risk score"[TIAB] OR "machine learning"[TIAB] OR "artificial intelligence"[TIAB] OR algorithm*[TIAB]) AND (triage[TIAB] OR escalation[TIAB] OR "rapid response"[TIAB] OR transfer[TIAB] OR "resource allocation"[TIAB] OR workflow[TIAB] OR timeliness[TIAB] OR delay*[TIAB] OR handoff*[TIAB]) ) AND ("2019/01/01"[PDAT] : "2025/10/18"[PDAT]) AND english[LANG]

**S1.2 Scopus**

**Database:** Scopus
**Year limits:** PUBYEAR 2019–2025 (implemented as PUBYEAR > 2018 AND PUBYEAR < 2026)
**Document type:** Article OR Review
**Language:** English
**Subject areas:** Medicine; Nursing; Health Professions; Computer Science
**Results (run date 30 Dec 2025):** 83 records

**Exact search string (copy/paste):**

TITLE-ABS-KEY(

("postpartum hemorrhage" OR "obstetric hemorrhage" OR eclampsia OR "hypertensive emergency" OR "hypertensive crisis" OR "maternal sepsis" OR "obstetric sepsis" OR ("early warning" AND (maternal OR obstetric* OR pregnancy OR postpartum)))

AND

("clinical decision support" OR CDS OR alert* OR "risk score" OR "machine learning" OR "artificial intelligence" OR algorithm*)

AND

(triage OR escalation OR "rapid response" OR transfer OR "resource allocation" OR workflow OR timeliness OR delay* OR handoff*)

)

AND PUBYEAR > 2018 AND PUBYEAR < 2026

**Applied Scopus UI filters (run date 30 Dec 2025):**

- Year: 2019–2025
- Document type: Article; Review
- Language: English
- Subject area: Medicine; Nursing; Health Professions; Computer Science

**S1.3 Record yield and deduplication**

PubMed yielded 722 records and Scopus yielded 83 records (total 805). After EndNote deduplication (Find Duplicates with manual verification), 779 unique records remained.
