# Supplementary material for: Equity at the point of care: auditing AI-supported resource allocation in obstetric emergencies
Source: Front Public Health. 2026 Mar 3;14:1774367. doi: 10.3389/fpubh.2026.1774367 (PMC12992295; doi:10.3389/fpubh.2026.1774367)
Supplement: Supplementary file 1 [file Supplementary_file_1.zip › Supplementary Box S3.DOCX]

**Supplementary Box S3. High-yield remediation (“debiasing”) toolbox mapped to MFAS links**

| Trigger / data provenance (T1 integrity)  • Lever: default vital-sign capture workflows; missingness alerts; provenance flags for unreliable timestamps.  • Verify: key-input missingness rate; trigger-time availability (T1 captured %); timestamp-lag distribution (charting delay).  • Owner: Health IT + Quality/Safety.  Response (T1→T2; actionability of alerts)  • Lever: one-click escalation coupled to an SLA and auto-paging; explicit alert ownership; monitor “acknowledged-no-action.”  • Verify: median (IQR) T1→T2; acknowledged-no-action rate; escalation completion within window.  • Owner: OB leadership / rapid response governance.  Resource-ready (T1→T4; blood/OR/ICU/transfer readiness)  • Lever: explicit cross-service priority rules (blood bank/OR/ICU); brief surge drills; override logging + review.  • Verify: time-to-resource-ready (T1→T4-* by resource); override rate + reasons; capacity-constraint flag frequency.  • Owner: Blood bank + OR/anesthesia + ICU leadership.  Closed-loop referral (acceptance and completion)  • Lever: named navigator/transfer-center ownership; live transfer traceability; escalation trigger if acceptance not secured within the window.  • Verify: acceptance-within-window rate; completion time (acceptance → departure/arrival, if captured); failure-mode mix (capacity vs escalation vs documentation).  • Owner: Transfer center / regional referral network governance. |
| --- |

**Abbreviations:**IQR, interquartile range; ICU, intensive care unit; IT, information technology; MFAS, Minimum Fairness Audit Set; OB, obstetrics/obstetric; OR, operating room; SLA, service-level agreement; T1, trigger timestamp; T2, response/assessment timestamp; T4, resource-ready timestamp.
